# A mixed methods approach to evaluating community drug distributor performance in the control of neglected tropical diseases

**DOI:** 10.1186/s13071-016-1606-2

**Published:** 2016-06-16

**Authors:** Fiona M. Fleming, Fred Matovu, Kristian S. Hansen, Joanne P. Webster

**Affiliations:** Schistosomiasis Control Initiative, Imperial College London, London, UK; School of Economics, Makerere University, Kampala, Uganda; Department of Global Health and Development, London School of Hygiene and Tropical Medicine, London, UK; Department of Infectious Disease Epidemiology, Imperial College London, London, UK; Department of Pathology and Pathogen Biology, Centre for Emerging, Endemic and Exotic Diseases (CEEED), Royal Veterinary College, University of London, Hertfordshire, AL9 7TA UK

**Keywords:** Neglected tropical diseases, Control, Integration, Community drug distributors, Mixed methods, Performance, Opportunity cost, Preventive chemotherapy

## Abstract

**Background:**

Trusted literate, or semi-literate, community drug distributors (CDDs) are the primary implementers in integrated preventive chemotherapy (IPC) programmes for Neglected Tropical Disease (NTD) control. The CDDs are responsible for safely distributing drugs and for galvanising communities to repeatedly, often over many years, receive annual treatment, create and update treatment registers, monitor for side-effects and compile treatment coverage reports. These individuals are ‘volunteers’ for the programmes and do not receive remuneration for their annual work commitment.

**Methods:**

A mixed methods approach, which included pictorial diaries to prospectively record CDD use of time, structured interviews and focus group discussions, was used to triangulate data on how 58 CDDs allocated their time towards their routine family activities and to NTD Programme activities in Uganda. The opportunity costs of CDD time were valued, performance assessed by determining the relationship between time and programme coverage, and CDD motivation for participating in the programme was explored.

**Results:**

Key findings showed approximately 2.5 working weeks (range 0.6–11.4 working weeks) were spent on NTD Programme activities per year. The amount of time on NTD control activities significantly increased between the one and three deliveries that were required within an IPC campaign. CDD time spent on NTD Programme activities significantly reduced time available for subsistence and income generating engagements. As CDDs took more time to complete NTD Programme activities, their treatment performance, in terms of validated coverage, significantly decreased. Motivation for the programme was reported as low and CDDs felt undervalued.

**Conclusions:**

CDDs contribute a considerable amount of opportunity cost to the overall economic cost of the NTD Programme in Uganda due to the commitment of their time. Nevertheless, programme coverage of at least 75 %, as required by the World Health Organisation, is not being achieved and vulnerable individuals may not have access to treatment as a consequence of sub-optimal performance by the CDDs due to workload and programmatic factors.

**Electronic supplementary material:**

The online version of this article (doi:10.1186/s13071-016-1606-2) contains supplementary material, which is available to authorized users.

## Background

In January 2012 the momentum to combat the neglected tropical diseases (NTDs) was accelerated with the London Declaration on NTDs [1]. Donors and endemic country governments, private sector leaders, pharmaceutical companies and multilateral organizations made a unique commitment for a close partnership to control or, in certain cases, eliminate ten NTDs by 2020, in alignment with World Health Organization (WHO) targets and the Roadmap on NTDs [2]. The roadmap is a guide to the implementation of policies and strategies to combat each of the NTDs and the extraordinary commitments towards these by each stakeholder.

As the primary implementers of mass preventive chemotherapy (PC) campaigns for NTDs in sub-Saharan Africa, village-elected Community Drug Distributors (CDDs) are critical stakeholders in achieving the Roadmap [3–5]. These stakeholders volunteer their efforts to perform multiple fundamental tasks which can include diagnosis, creating census records, treatment and reporting. If these individuals, who are trusted and respected within their communities and whom essentially act as the front-line health facility, are not supported adequately then entire disease control programmes could be in jeopardy.

In Uganda, where all 112 districts are endemic for one or more NTDs [unpublished data, Uganda MoH Master Plan for the control NTDs (2011–15)], there is an overall health worker deficit of approximately 80 % [6] and only approximately 49 % of the population live within five kilometres from a health facility [7]. ‘Task shifting’, where tasks are delegated to less specialised health workers, including community volunteers, and Village Health Teams (VHT) have been introduced by the Ugandan Ministry of Health to bridge these gaps [7, 8]. The CDDs are part of the VHT, where the structure exists, and these individuals play a pivotal role in the implementation and success of the country’s integrated NTD Programme. In Uganda, stand-alone NTD control programmes existed which were then integrated, or co-implemented, from 2007 onwards to reduce duplication of resources and costs [9]. The stand-alone programmes also used CDDs for drug distribution and, in many cases, the same individuals participate under the current integrated NTD Programme.

There are several conflicting views in the literature as to how integration of several control interventions impacts community volunteers such as CDDs. One view is that co-implementation of interventions can effectively make it easier for community volunteers, because they only have one multi-layered intervention to be responsible for which results in increased community volunteer efficiency, reduced reporting requirements, increased intervention utilisation as well as reduced time and transportation costs [10–12]. In contrast, by requesting community volunteer to be responsible for an intervention with multiple and additional services, there is the view that they will become overburdened, resulting in increased time and a subsequent decline effect in their performance [13, 14].

Diaries have been used in many contexts as a research tool to monitor daily experiences and behaviours which are expected to change over time. Events that are recorded can be as wide-ranging as the ‘high-risk’ behaviours of commercial sex workers to that of the incidence of colds or household consumption and expenditure [15–17]. The benefit of using diaries for data collection is that the information is recorded in real time which greatly minimises recall bias. Furthermore, in low-income countries where many of the population are illiterate, pictorial diaries have been shown to be a particularly effective tool for measuring activities, such as, expenditure activities and daily frequency and consistency of stools [18–20]. Although there is substantial literature about the CDDs roles in community-directed treatment with ivermectin (CDTI) for onchocerciasis control and in trachoma and schistosomiasis control [3–5], there is currently no evidence of the CDDs role in integrated PC (IPC). Furthermore, although diaries and work logs are used extensively in research, there are no published data which refers to pictorial diaries recording information on daily activities of community volunteers in mass treatment programmes.

The aim of the current study was to determine the role and the work burden of the CDD in the Ugandan integrated NTD Programme which delivers IPC on an annual basis. This was achieved using a mixed methods approach including a Pictorial Diary (PD) which was designed to collect daily prospective data on the time spent by CDDs on both their routine activities and NTD activities. By recording time spent on NTD activities and assigning a monetary value the opportunity costs of CDD time were determined. CDD performance was estimated from a validation IPC coverage survey. Additionally semi-structured interviews, questionnaires and focus group discussions (FGD) captured qualitative and socio-demographic data. The results of this study can be used by those responsible for implementing health interventions using community volunteers. Also of importance, the evidence can inform those funding and donating drugs to large-scale NTD Programmes on how the partner countries are contributing to these community-based programmes.

## Methods

### Study design

A prospective longitudinal survey was designed which used a mixed methods approach to collect both quantitative and qualitative data on the CDD contribution to the Ugandan NTD Programme. The CDDs were selected across four randomly selected districts. Districts were stratified according to the number of drug delivery rounds a CDD would have to carry out in their community over an annual mass treatment campaign. These were either one, two or three delivery rounds depending on the underlying endemicity of NTDs and required drug package within the district. Kamuli district represented three delivery rounds ivermectin (IVM) and albendazole (ALB) for lymphatic filariasis, praziquantel (PZQ) for schistosomiasis, azithromycin (ZIT)) for trachoma, Mayuge district (PZQ + ALB, ZIT) and Yumbe (IVM + ALB, PZQ), where IVM was also for treatment for onchocerciasis, were representative of two rounds and Pallisa (IVM + ALB) was selected from the stratum with one delivery round.

Across the four districts a sample of 64 CDDs was sufficient for both the depth of information from repeat engagement with the participants (using PDs), as well as breath of information across variables such as number of deliveries, distribution method and number of households served to allow for generalizability of the findings [21] and was in-line with other similar studies [22–24]. Each village has two CDDs which it elects to take part in the NTD Programme (A. Onapa, RTI International pers comm.), thus 32 villages were selected. Decisions on methods for distribution of tablets tend to be made, and so similarities occur, at the sub-county level or parish level and so for each of the four districts, multi-stage randomisation was used to select two sub-counties (8 sub-counties) and from these, two parishes (16 parishes) and finally from each parish, two villages were selected (32 villages). The total population of the study areas was an estimated 34,615 with approximately 5,724 households, thus 89 households would be served by each CDD (Ugandan Bureau of Statistics, www.ubos.org). The CDDs were enrolled into the study at least two weeks prior to the beginning of NTD Programme activities in October 2008, until June 2009 when programme activities for which they were involved, had been completed.

### CDD role in the NTD programme

Distribution mechanisms for IPC through the Ugandan NTD Programme are community-based and school-based. CDDs are elected by their communities or requested by their local councillors to participate in the programme. After receiving training from sub-district health personnel, the CDDs ‘volunteer’ their time to sensitise and mobilise communities about treatment for the NTDs and subsequent health benefits. Following this they distribute drugs to the eligible target population either from a focal point such as a church, health clinic, trading centre or by the CDD moving from door to door. Following distribution CDDs write coverage reports based on their treatment registers and submit the results and any remaining drugs to the nearest health unit.

### Data collection

Data collection was carried out in teams all trained by the study investigators and an experienced social scientist from Makerere University, Kampala. All interviews and FGD were held in the relevant local languages for the study and pre-testing districts: Lusoga for Kamuli and Mayuge districts, Lugwere in Pallisa district, Luganda in Mukono district (for pre-test) and Aringa Lugbara in Yumbe district. Data collection comprised of four components described herewith.

#### Pictorial diaries

The first component determined how CDDs conducted their days, in terms of daily routines and during NTD treatment campaigns. Two FGDs were held and CDDs were asked to recount their daily activities from when they awoke to when they went to bed until no new activities were mentioned. The CDDs were then asked to describe what symbols or pictures they thought might best represent these daily and routine activities. Meanwhile, two Ugandan artists were sketching these symbols and pictures. Subsequently the CDDs were requested to review the symbols and pictures developed by the artists and identify which activities they represented. Where the pictures were identified incorrectly or were not clear the CDDs were asked to describe how they could be improved to be more recognizable.

The pictures were further refined and FGD were conducted across three districts to draw out any daily routine and NTD activities not previously mentioned, highlight any seasonal variations in routines and to review the set of illustrations to suggest any improvements for clarity. CDDs were also asked to describe how they measured their time, for instance, what tools or features they might use to identify the time of day and how they split the day. The most common methods of telling the time used by the CDDs were radio programmes, especially the news; mobile phones; clocks in their houses; Muslim call to prayers; and for those who lived near schools, the school bell (the banging of a wheel) at different times during the day. In each district the CDDs split the day into morning, afternoon, evening and night with only minor differences at what time they began and ended. All CDDs reported that they used ‘Swahili’ time throughout the day, where 7 a.m. is 1 o’clock, 8 a.m. is 2 o’clock and so on. Finally, during the FGD, the CDDs were asked about the feasibility of filling a diary of their activities on a daily basis.

Subsequently, two pictorial diary formats were developed. The first broke each day into hours e.g. 1–2, 2–3, 3–4 and would require the CDD to identify what activities they were involved in hour by hour and tick in the relevant boxes. This was called the ‘hour’ PD. The other format known as the ‘time’ diary, had only one column for each day and would require them to mark down the number of minutes spent doing an activity each time they carried it out in that day. Both formats were pre-tested in Mukono district chosen for its proximity to Kampala and because it was still a rural environment with CDDs involved in the NTD Programme. Four villages were chosen and seven, out of a potential eight, CDDs were at their homes. The CDDs were then randomly given the ‘hour’ or the ‘time’ PD and were talked through each of the pictures and asked to interpret what routine or NTD activity they saw. An explanation of how the PD works and the purpose of it were given and the CDD asked if they consented to record their time in the diary for one week. On follow-up the ‘hour’ PD (Additional file 1: Figure S1) was revealed to be easier to use, with fewer inconsistencies, than the ‘time’ PD, with a few minor amendments to the pictures highlighted. CDDs indicated they would be happy to fill the PD from between two months to one year, and all felt that the time it took to complete on a daily basis was not a burden.

Final adaptations were made to the ‘hour’ PD and an *aide mémoire* was developed (see Additional file 1). In the four selected study districts, the study teams set up the PDs with the 64 CDDs at their homes. During the initial visit CDDs were given an explanation to the purpose of the diary and how to complete it, including a practice for the previous day’s activities. The CDDs were followed-up one week later to check progress on PD entries by reviewing the activities recorded hour by hour for each day. Issues encountered, for example, not remembering exactly where a certain activity be marked, were discussed in detail and all the pictures reviewed to ensure the CDD fully understood each one. The CDDs were visited again two weeks after the previous visit where accuracy in PD completion was reviewed and a repetition of the pictures and how to fill the PD were given if there was insufficient clarity. CDDs were subsequently visited every two weeks, during which PDs were reviewed and semi-structured interviews were held after each NTD activity had been completed.

#### CDD semi-structured interviews and focus group discussions

The second and third components explored the CDDs participation in the NTD Programme through semi-structured interviews and FGDs. The interviews explored a series of background questions pertaining to socio-demographic characteristics, past and present involvement in CDD activities for NTDs and involvement in other health interventions. A set of pre-defined closed and open-ended questions were asked after each NTD Programme activity e.g. training, collecting drugs and mass drug administration (MDA) of PC drugs. These interviews focused on the CDDs role in and experience of the activity. Once all the NTD Programme activities were complete self-reported performance, motivation and an attitude scale were assessed through a final interview. Motivation in addition to overall experiences of and perceived responsibilities within an NTD Programme were explored further in an FGD held in each sub-county with between six and eight CDD participants.

Interview schedules for the semi-structured interviews and topic guides for FGD were developed. The tools were translated and back-translated, then piloted and adapted accordingly. Interviews and FGD were conducted in local language by the trained, local research assistants. The interviews were held at each CDD residence alongside the PDs. The FGD were conducted in convenient public places identified by the CDDs. All sessions were recorded, transcribed, translated, and back translated to ensure accuracy and quality.

#### Post-MDA drug coverage survey

Effectiveness of NTD Programmes is measured by performance indicators, the main being coverage of at-risk and eligible populations [9, 25]. Reported treatment coverage originates from each CDDs treatment register. CDD treatment registers were retrospectively assessed for accuracy of total treated for each drug against two recounts of CDD entered numbers, using a calculator by the study investigators. In all cases the total numbers recorded by the CDDs were inaccurate by more than 12 % with the majority being overestimations. Validated treatment coverage was obtained by including the study villages in a concurrently run independent post-MDA survey. The methodology for this fourth component, a multistage cluster sample coverage survey, is described elsewhere [26].

### Data management and analysis

CDD time data from the PDs were double-entered into a customised Microsoft Excel database (Microsoft Corp. Seattle, WA, USA). Mean number of minutes were calculated for all routine daily activities and for each NTD Programme activity. Minutes were then converted into hours. Working days were based on an eight-hour day and working weeks were based on five working days i.e. 40 h were in a working week. To calculate the annual proportion of time spent on NTD Programme activities 246 working days was used.

As part of the national NTD Programme all CDDs received, regardless of whether they were part of the study, a financial stipend of 2,000 to 4,000 Ugandan shillings (USh) (US$0.89–1.79) when they attended training. No other remuneration was provided. From a societal perspective, CDDs incur an opportunity cost for participating in the NTD Programme as they are unable to perform their normal activities. Opportunity costs include the value forgone by the CDDs time not working in their shamba (gardens), doing casual labour or carrying out retail business. CDDs volunteer time was valued with a base case of 6,000 USh per day (US$2.70) which was the value of local casual labour wages [27]. This was equivalent to an hourly rate of 750 USh (> US$0.34) based on an eight hour working day. The minimum wage on the Government of Uganda salary scale (4,193 USh or US$1.95 per day) and GNI *per capita* (7,931 USh or US$3.70 per day) were also used. All prices were adjusted for inflation over time using the GDP implicit price deflator and expressed in US$ 2010 prices (IMF, 2008 http://www.imf.org/external/pubs/ft/weo/2008/01/weodata/index.aspx).

Data were tested and approved for normality and all statistical analyses were carried out using STATA 11.2 (StatCorp LP, TX, USA). Time variables which were not non-normally distributed were transformed to the logarithmic scale. PD time data were analysed using a paired *t*-test to compare means of daily routine activities between days with, and without, NTD activities, during the same period. One-way analysis of variance (ANOVA) and simple linear regression were used to test for differences in the mean times between different levels of the independent variables, such as, administration level, distribution method, population and number of households served, length of tenure as a CDD, the number of drug deliveries (one delivery against two deliveries, one delivery against three deliveries, two deliveries against three deliveries), and socio-demographic variables. Mixed model linear regression was performed to determine if the total hours spent by the CDDs on the NTD Programme, whilst controlling for confounding and clustering at the parish level, were still significantly associated with those independent variables which had been identified during simple linear regression.

Treatment coverage data were entered into EPI Info (Version 6.04, USA CDC, Atlanta, GA). Mean coverage were calculated using the survey function in STATA which takes into account the clustered sampling design. Simple linear regression was also used to look at which variables were associated with treatment coverage. For both time and coverage, multiple linear regression included all variables that were found to be statistically associated with the outcome variable to adjust for the effects observed by these variables.

For the semi-structured and FGD quantitative and qualitative data, a coded scheme was developed by pre-defined topics together with themes emerging from the data using the qualitative data analyses software NVIVO (Version 9. QSR International, Doncaster, Australia).

## Results

### Participant characteristics

A total of 58 CDDs out of 64 participated in the study to completion. Reasons for the six non-participators were in some cases due to the election of only one CDD and in others, dropping out of the NTD Programme after training but before MDA. Reasons for drop out were: not having enough time to carry-out NTD Programme activities and migration for fishing purposes. Socio-demographic and study characteristics are summarised in Table 1. A third of participants were female and average age of all participants was 36 years (range 21–64). Of the CDDs, 98 % were married and 28 % had completed only primary education; 72 % had completed primary and, at least, three years of secondary school education. In addition, 40 CDDs reported their main occupation was subsistence farming, five were in retail and six CDDs were market traders. CDDs distributed the drugs either door to door, i.e. visiting each village house and treating all members present (47 %), or from a focal point such as a health centre, market place, school (53 %). The average population size served by a CDD was 497 (range 161–1437) and approximately 90 households (range 31–313). The length of tenure for the CDDs in the NTD Programme and, if applicable prior stand-alone control programmes for NTDs, was one to five years including the year of study.

**Table 1:** Socio-demographic and study characteristics; Mean time (hours) per day spent on routine activities carried out not-during NTD Programme and during NTD Programme, and on NTD Programme activities according to descriptive characteristics for the 58 CDDs

	Number (%)	Routine activities		NTD Programme activities mean hours (95 % CI)
		Not during-NTD Programme mean hours (95 % CI)	During-NTD Programme mean hours (95 % CI)	
Overall	58 (100)	16.87 (16.67–17.06)	12.50 (11.97–13.04)	105.99 (83.80–128.19)
Gender				
women	18 (31.03)	16.89 (16.47–17.30)	12.43 (11.13–13.72)	88.01 (59.30–116.73)
men	40 (68.97)	16.86 (16.65–17.08)	12.54 (12.01–13.07)	114.08 (84.75–143.42)
Age groups (mean 36 years; range 21–64 years)				
< 30	11 (18.97)	16.63 (16.28–16.98)	12.61 (11.56–13.67)	119.56 (64.91–174.22)
30–34	15 (25.86)	17.06 (16.71–17.41)	12.84 (11.93–13.74)	83.37 (68.07–98.67)
35–49	22 (37.93)	16.84 (16.48–17.21)	12.25 (11.14–13.36)	94.77 (65.70–123.84)
50 ≤	10 (17.24)	16.92 (16.48–17.36)	12.44 (11.53–13.35)	149.69 (59.39–239.98)
Marital status				
Married	57 (98.28)	16.87 (16.67–17.06)	12.52 (11.98–13.07)	105.97 (83.39–128.56)
Single	1 (1.72)	16.91 (na)	11.22 (na)	107 (na)
Education level				
Primary	16 (27.59)	16.66 (16.25–17.07)	12.24 (10.79–13.69)	87.19 (47.18–127.19)
Secondary & above	42 (72.41)	16.95 (16.74–17.17)	12.60 (12.09–13.11)	113.16 (86.61–139.70)
Occupation				
Subsistence farming	40 (68.97)	16.82 (16.56–17.08)	12.47 (11.78–13.16)	106.2 (79.07–133.31)
Retail	5 (8.62)	16.85 (16.51–17.19)	13.07 (11.83–14.32)	69.26 (38.13–100.40)
Market trader	6 (10.34)	17.26 (16.88–17.65)	11.94 (10.75–13.13)	130.84 (69.64–192.04)
Fisherman	1 (1.72)	17.09 (na)	9.56 (na)	94.17 (na)
Housewife	3 (5.17)	16.82 (15.96–17.68)	13.29 (10.31–16.28)	55.31 (8.35–102.26)
Religious leader	2 (3.45)	16.66 (16.29–17.03)	12.34 (11.88–12.80)	202.91 (102.99–508.80)
Teacher	1 (1.72)	16.85 (na)	15.09 (na)	102.57 (na)
Distribution method				
Door to door	27 (46.55)	16.95 (16.70–17.20)	12.61 (11.89–13.33)	103.67 (78.63–128.72)
Focal point	31 (53.45)	16.80 (16.51–17.09)	12.41 (11.61–13.20)	108 (72.28–143.74)
Number of households served (mean 90; range 31–313)				
0–69	24 (41.38)	16.93 (16.56–17.29)	11.72 (10.69–12.74)	79.58 (65.04–94.12)
70–99	16 (27.59)	16.92 (16.63–17.20)	13.13 (12.44–13.81)	72.09 (51.14–93.05)
100≤	18 (31.03)	16.76 (16.45–17.07)	12.99 (12.28–13.71)	171.33 (115.41–227.27)
Population size served (mean 497; range 161–1437)				
0–399	26 (44.83)	16.97 (16.67–17.26)	11.93 (10.95–12.91)	75.43 (63.80–87.05)
400–599	16 (27.59)	16.60 (16.23–16.97)	12.86 (12.18–13.53)	71.66 (51.77–91.55)
600 ≤	16 (27.59)	16.98 (16.64–17.32)	13.07 (12.27–13.88)	189.99 (131.54–248.45)
Length of tenure as a CDD				
NTDCP only - 1 year	9 (15.51)	17.05 (16.50–17.60)	13.06 (11.89–14.23)	105.28 (64.81–145.75)
NTDCP only - 2 years	33 (56.90)	16.77 (16.49–17.04)	12.04 (11.31–12.76)	88.71 (67.05–110.37)
NTDCP + SA 3–6 years	16 (27.59)	16.98 (16.72–17.25)	13.14 (12.16–14.12)	142.03 (81.05–203.02)

*Abbreviation*: *na* not available

## CDD time allocation

### Mean hours on routine activities

Total PD days analysed were restricted to those from when the first NTD programme activity, to when the last NTD Programme activity, took place. Data on CDD activities were analysed from 6,825 days of which 878 days were time spent on NTD activities. Mean hours spent on routine activities, on those days without NTD Programme activities, for all of the 58 participants were 16.87 h (95 % CI: 16.67 to 17.06). The mean hours spent on routine activities during days with NTD Programme activities were 12.50 h (95 % CI: 11.97 to 13.04). Table 1 shows the variance observed around these means across the participant characteristic variables. No differences between the overall mean hours and the mean hours across the participant characteristic variables were seen for either routine activities grouping. The routine activities which were identified by the CDDs and included in the PDs are listed in Table 2. The total time spent on daily routine activities was significantly lower during-NTD Programme than when programme activities were not being carried out (12.50 h, 16.87 h, *t*_(57)_ = 17.79, *P* < 0.001). A significant reduction in time spent on daily activities was observed between not during-NTD Programme to during-NTD Programme for working time in the shamba (2.78 h, 1.87 h, *t*_(57)_ = 6.37, *P* < 0.001), preparing and eating meals (2.14, 1.81, *t*_(57)_ = 5.41, *P* < 0.001), relaxing and socialising (3.33 h, 1.85 h, *t*_(57)_ = 11.96, *P* < 0.001) and family-time (2.03 h, 1.78 h, *t*_(57)_ = 3.66, *P* < 0.001).

**Table 2:** Mean hours, per day, spent on daily routine activities not during and during the NTD Programme

Activity^a^	Not during-NTD Programme mean hours (SD)	During-NTD Programme mean hours (SD)	Difference	*P-* value
Bathing	1.30 (0.37)	1.23 (0.37)	-0.07	0.003
Praying	1.58 (0.86)	1.46 (0.72)	-0.12	0.035
Preparing children for school	0.13 (0.15)	0.11 (0.14)	-0.02	0.150
Work in the shamba	2.78 (1.09)	1.87 (1.17)	-0.91	< 0.001
Preparing and eating meals	2.14 (1.48)	1.81 (1.89)	-0.33	< 0.001
Tending to animals	0.89 (0.82)	0.62 (0.63)	-0.27	< 0.001
Relaxing and socialising	3.33 (1.46)	1.85 (1.19)	-1.48	< 0.001
Household chores	0.69 (0.65)	0.48 (0.47)	-0.21	< 0.001
Family time	2.03 (1.21)	1.78 (1.16)	-0.25	< 0.001
Business	1.50 (1.79)	0.95 (1.50)	-0.55	< 0.001
Non-NTD health interventions^b^	0.49 (0.62)	0.34 (0.61)	-0.16	0.043
Total time	16.87 (0.73)	12.50 (2.04)	-4.37	< 0.001

^a^sample size was 58 CDDs for each activity

^b^non-NTD health interventions included CDD involvement in home-based management of fever for malaria, community mobilisation for immunisation campaigns and health educator of HIV/AIDS or good hygiene practices

### Mean hours on NTD Programme activities

The CDDs spent an average of 105.99 h (95 % CI: 83.80–128.19) or 13.31 work-days (range 2.65–56.93 days) on NTD Programme activities over one year (Table 1). Under NTD Programme activities, significant differences were seen between the overall mean and the mean hours spent across the categories of households served (*F*_(2,55)_ = 8.4, *P* < 0.001) and population served (*F*_(2,55)_ = 12.8, *P* < 0.001). In the study design, districts were selected to represent the different number of deliveries that would take place over one IPC campaign. However, in Mayuge district due to an insufficient supply of drugs not all target areas received the necessary PC. Subsequently one of the sub-counties had only one delivery round for ZIT and not a second for PZQ and ALB as planned. Analyses were subsequently broken down by number of delivery rounds and not district. One delivery of PC drugs was borne by 23 CDDs, two deliveries by 22 CDDs and three deliveries by 13 CDDs (Table 3). When split by delivery, the mean time spent on the NTD Programme activities by the CDDs increased from 83.30 h (one delivery), to 112.09 h (two deliveries) and to 135.81 h (three deliveries), with a significant difference between one and three deliveries (*t*_(35)_ = 2.69, *P* = 0.01).

**Table 3:** Mean hours, per IPC campaign spent on NTD Programme activities by the number of delivery rounds borne by the CDD

	Number of deliveries			
Activity	One [hrs] (SD)^a^	Two [hrs] (SD)	Three [hrs] (SD)	*P-*value*
				< 0.001
				1d – 2d, 0.21
				2d – 3d, 0.02
Collecting drugs	6.42 (16.99)	6.74 (8.06)	16.87 (17.10)	1d – 3d, 0.001
				0.02
				1d – 2d, 0.19
				2d – 3d, 0.26
MDA	37.94 (37.03)	57.10 (51.87)	77.10 (73.04)	1d – 3d, 0.01
				0.09
				1d – 2d, 0.73
				2d – 3d, 0.03
Health education & mobilisation	16.03 (19.51)	21.09 (34.50)	9.05 (8.63)	1d – 3d, 0.08
				0.35
				1d – 2d, 0.23
				2d – 3d, 0.02
Registration	13.18 (17.57)	13.04 (24.87)	24.04 (20.81)	1d – 3d, 0.19
				0.93
				1d – 2d, 0.26
				2d – 3d, 0.12
Reporting	4.28 (5.07)	6.01 (4.17)	2.12 (2.68)	1d – 3d, 0.61
				0.28
				1d – 2d, 0.06
				2d – 3d, 0.26
Training	5.45 (4.07)	8.13 (7.86)	6.63 (3.27)	1d – 3d, 0.35
				0.02
				1d – 2d, 0.32
Total	83.30 (57.88)	112.09 (104.98)	112.09 (104.98)	2d – 3d, 0.16
Range^b^	25.67–243.50	21.17–455.45	21.17–455.45	1d – 3d, 0.01
Number of CDDs	23	22	13	

^a^Standard deviations (SD) of the mean are given in the parenthesis

^b^The range in total hours for each delivery is highlighted in the bottom row of the table

*Overall *P*-value between the number of deliveries and then between one delivery (1d) and two deliveries (2d), two deliveries (2d) and 3 deliveries (3d) and one delivery (1d) and 3 deliveries (3d)

**Fig. 1:**
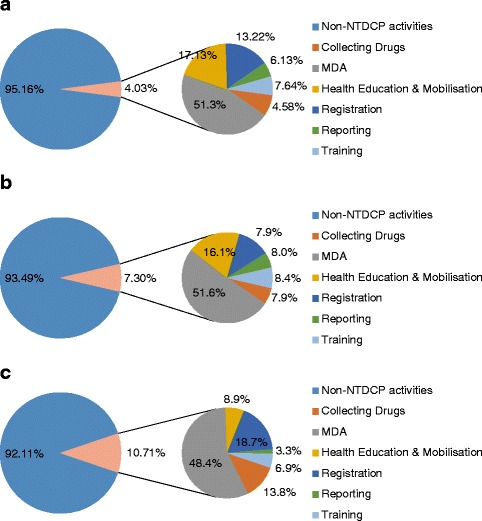
Proportion of time spent on NTD Programme activities by CDDs during an average working year for (**a**) one, (**b**) two and (**c**) three delivery rounds required during an IPC campaign

Table 3 shows the mean time on each NTD Programme activity per delivery and Fig. 1a-c show the proportion of overall time spent on routine activities and time spent on each NTD Programme activity by number of deliveries during the implementation period of the NTD Programme. There were no statistically significant differences in hours spent by a CDD conducting either one or two deliveries for each of the NTD Programme activities. The hours spent collecting drugs from the health units was significantly higher between two and three (*t*_(34)_ = 2.54, *P* = 0.02), and one and three deliveries (*t*_(35)_ = 3.50, *P* = 0.001), whereas with distributing the drugs during the IPC campaign there was a statistically higher number of hours spent between one and three deliveries only (*t*_(35)_ = 2.66, *P* = 0.01). Both the conducting of health education and mobilisation (*t*_(34)_ = -2.32, *P* = 0.03), and registration (*t*_(34)_ = 2.39, *P* = 0.02) required a statistically different number of hours between those CDDs which had to deliver two rounds of PC as compared to those who had to deliver three rounds of PC, with the former activity actually reporting less hours with increasing number of deliveries.

The number of households (*t*_(57)_ = 3.51, *P* = 0.001) and village population size (*t*_(57)_ = 4.36, *P* < 0.001) served by a CDD were also significantly associated with the overall time spent of NTD Programme activities. The amount of time CDDs spend on NTD Programme activities was not statistically different between the distribution methods of door to door or from a focal point (*t*_(57)_ = -0.27, *P* = 0.79). Neither were any socio-demographic variables such as gender (*t*_(57)_ = -1.44, *P* = 0.16), age (*t*_(57)_ = 0.44, *P* = 0.66), marital status (*t*_(57)_ = 0.36, *P* = 0.72), education level (*t*_(57)_ = 1.53, *P* = 0.13), occupation (*t*_(57)_ = 0.19, *P* = 0.85) and length of tenure as a CDD (*t*_(57)_ = 1.31, *P* = 0.20). Mixed model linear regression (Table 4) indicated that, per CDD, number of deliveries and population served were still significant determinants of time spent on NTD programme activities when controlling for confounding and clustering seen at the parish level.

**Table 4:** Mixed model linear regression to test associations between independent variables on total hours spent on the NTD Programme by CDDs whilst controlling for confounding and clustering at parish level

Dependent variable: total hours spent on NTD Programme by the CDDs				
Independent variable	Coefficient	Standard error	*Z*	*P*-value
Number of deliveries per CDD	0.228	0.112	2.03	0.042
Population served per CDD	0.002	0.001	2.68	0.007
Number of households per CDD	-0.007	0.004	-1.58	0.114
Constant	3.474	0.259	13.41	< 0.001

### CDD performance

District level reported treatment coverage and survey treatment coverage for specific drug combinations in the study districts are shown in Table 5. Median survey coverage and confidence intervals take into account the cluster design effect. The reported therapeutic and programme coverage both lie within the 95 % confidence interval for the validated survey coverage for only PZQ + ALB in Mayuge district [35 % eligible *vs* 36 % survey (95 % CI: 32–30 %)] and IVM + ALB in Yumbe district [74 % at-risk *vs* 73 % survey (95 % CI: 70–76 %)].

**Table 5:** District reported coverage and validated survey coverage data from national post-MDA drug coverage survey

District	Drug packages	Coverage					
		Reported			Survey		
		Treated	Therapeutic coverage (%)^a^	Programme coverage (%)^b^	Median (%)	*n*	95 % CI
Kamuli	ZIT	396,700	61	63	36.73^c^	1078	33.85–39.61
	IVM + ALB	508,573	78	97	52.71^c^	1089	49.73–55.67
	PZQ	29,470	75	94	57.39^c^	1089	54.45–60.33
Mayuge	ZIT	182,763	44	46	13.07^c^	1094	11.07–15.07
	PZQ + ALB	37,666	28	35	36.42	1102	32.43–40.42
Yumbe	IVM + ALB	295,179	74	93	72.76	984	69.97–75.55
	PZQ	278,670	70	88	77.16^c^	972	74.51–79.80
Pallisa	IVM + ALB	386,859	92	96	62.59^c^	1096	59.72–65.46

^a^Therapeutic coverage is the (Number of individuals ingesting the PCT drugs for a specific disease in an endemic country/district etc./Total number of individuals in the country/district etc., all at risk of infection) × 100 [44]

^b^Programme coverage is the (Number of individuals in the target population ingesting the PCT drugs in [x] endemic area/All the eligible individuals targeted for treatment in the [x] endemic area) × 100 [44]

^c^reported at-risk or eligible coverage lies out-with the validated survey coverage 95 % confidence interval

**Fig. 2:**
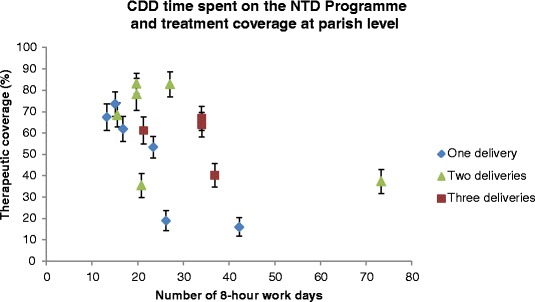
Mean number of 8 h working days spent carrying out NTD Programme activities by the CDDs at the parish level) plotted against the mean highest validated treatment coverage achieved by each CDD in the parish. Colours represent how many deliveries were carried out in each parish by the CDDs. Upper and lower 95 % confidence intervals are shown by the error bars around each plot

CDD performance was specifically measured by village-level survey treatment coverage. Village-level programme coverage was significantly associated with number of deliveries (*t*_(31)_ = 2.03, *P* = 0.05) and negatively associated with the number of hours spent on NTD Programme activities (*t*_(31)_ = -2.16, *P* = 0.04). When controlled for confounding, only the latter variable continued to be significantly associated with programme coverage (*t*_(31)_ = -2.54, *P* = 0.02). Figure 2 also indicates that when treatment coverage and time spent by CDDs on the NTD Programme are analysed together, lower programme coverage was achieved with increasing time spent on NTD Programme activities, at the parish level.

### CDD motivation and attitude

At study inception, when asked about their motivation for taking part in the NTD Programme as a volunteer, 82 % of CDDs responded that they participated to reduce sickness in their community. Alternative leading reasons given were, their recognition as a ‘Musawo’ or doctor (61 %), to support government health programmes (57 %), to reduce stigma for affected individuals (47 %) and to increase knowledge on health issues (36 %). The same reasons were given by 83 % of volunteers when later asked, if, and why, they would commit again to being a volunteer for the NTD Programme. During the FGD such responses were elucidated by the CDDs:

*“I was motivated by the trust that the people put in me. They thought I would serve them truthfully, I had to accept to work for them, not to let them down.”* Kagulu, Kamuli

*“What motivated me to do this job is the magnitude of disease that is in my community. I knew that if I did the work of distributing drugs well, then my people will become healthier.”* Romogi, Yumbe

In total 44 CDDs (76 %) were involved in other health interventions such as health mobiliser, sanitation and hygiene education, home-based delivery of malaria treatment, bed-net distribution, HIV/AIDS and TB awareness training, and polio and immunisation campaigns. CDDs responded that they were less motivated (63 %) to carry out their NTD Programme activities in comparison to their other health interventions with major reasons being, HIV/AIDs, malaria and TB were major public health concerns in the area and cause mortality, NTDs are less known in the community, financial incentives were given for other health interventions and NTD treatment required more travelling around the community and frequent collection of drugs from health centres. Of the remaining CDD responses 25 % were equally motivated and 12 % were more motivated. Below are several quotes from the CDDs with regards to their motivation for the NTD Programme.

*“If they would give us some money it would motivate us to work with more interest. But we forego our own activities to work for free, while people are assuring us that we are being paid.”* Petete, Pallisa

*“Since it’s voluntary, we put aside our personal duties and do NTD work which is free. When we reach home from sensitization they need money for food, school fees and sometimes wives think (NTD) its paying. So, trouble can come up in the family.”* Kei, Yumbe

An excerpt from the Attitude Scale asked at the final CDD interview is shown in Table 6. This presents the CDD participants responses pertaining to health activities in the communities and motivation. In summary, CDDs agreed that by being involved in concurrent health activities, made it easier to mobilise communities for the NTD Programme and that their overall performance was better. Due to limited health facilities in the communities the CDDs regarded their work as enhancing and supporting health services. Nevertheless, the CDDs strongly agreed that they required monitoring and supervision by health staff. Finally the CDDs disagreed that the support given by the community was enough motivation and the majority agreed that further support was required from the Ministry of Health (MoH).

**Table 6:** Extract from the Attitude Scale in where CDDs provided a response to the statements asked in relation to their participation and motivation in the NTD Programme

Statements	Strongly agree	Agree	Indifferent	Disagree	Strongly disagree
CDDs are capable of handling several health activities at the same time	7 % (4)	79 % (46)	–	7 % (4)	7 % (4)
When a CDD is involved in other health-related activities, this helps in mobilizing the community for the NTD MDA	50 % (29)	50 % (29)	–	–	–
The work of the CMD has improved since they got involved in additional health activities (non-NTD activities)	–	100 % (58)	–	–	–
To be effective, a CDD should not be involved in other health-related activities (non-NTD activities)	–	–	–	100 % (58)	–
Because there are few health services in the communities, CDDs have to carry out health activities (NTD and non-NTD)	–	79 % (46)	–	7 % (4)	14 % (8)
The involvement of CDDs in health activities enhances health services in this community	7 % (4)	93 % (54)	–	–	–
The involvement of CDDs in NTD activities requires frequent monitoring and supervision by health staff	57 % (33)	36 % (21)	–	7 % (4)	–

### Opportunity costs of CDD time

The opportunity cost of CDD time was estimated using the time spent on NTD Programme activities combined with values for the local labour wage, the minimum wage on the national salary scale and GNI *per capita*. The results are presented in Table 7 and demonstrate the mean opportunity cost for being involved in the NTD Programme for a CDD is US$35.71, US$25.77 and US$49.09, respectively, for each labour value. The number of hours increases with increasing number of deliveries and consequently, as do opportunity costs of the CDD participating in the NTD Programme. With proportion of annual income as a benchmark, the opportunity cost of CDD involvement in the NTD Programme increases from 4.23 %, for one delivery, to 5.69 % for two deliveries and to 6.91 % for three deliveries.

**Table 7:** Mean time (hours) and [days] spent by CDDs and estimated opportunity costs of CDD time spent on NTD Programme activities using different salary values

Number of delivery rounds	Mean time on NTDCP (hrs) [days^a^]	Labour wage (USD$^b^)	Minimum national wage (USD$^b^)	GNI *per capita* (USD$^b^)	Proportion of annual income (%)
One delivery	83.30 [10.4]	28.06	20.25	38.58	4.23
Two deliveries	112.09 [14.0]	37.76	27.25	51.92	5.69
Three deliveries	135.81 [17.0]	45.75	33.02	62.90	6.91
Mean total	105.99 [13.3]	35.71	25.77	49.09	5.41
Annual income^c^		666.03	478.49	911.57	100

^a^8 h working day

^b^In 2010 US$ prices

^c^estimated if 246 working days per year

All 58 (100 %) of the CDDs participating in the study responded that the monetary incentive they currently receive was not satisfactory. For all annual NTD Programme activities CDDs receive an allowance for lunch and transport only during training which varies between districts but on average is US$1.80. This payment is regardless of the number of drug delivery rounds to be made. A NTD Programme t-shirt (US$1.86) was received by 32 % of the participants in the study. Calculation of out-of-pocket expenses incurred by the CDDs, reported during interviews were approximately US$2.78 per person (range US$1.32–US$8.61). These expenses were predominantly for transportation to collect drugs from the health units and for drug distribution, as well as for lunch whilst carrying out NTD Programme activities that required substantial travelling such as, registration, drug collection and treatment.

When asked what financial or non-financial reward would be compensation for NTD Programme duties 67 % of CDDs felt a bicycle would facilitate programme activities and 42 % felt that a uniform was necessary to clearly be identified as an NTD Programme implementer. Other non-financial rewards suggested were t-shirts (by those who had not received), bags, hats, boots and waterproof coats with the programme logo and certificates. All felt that an annual financial reward should be received of approximately US$9–US$18. The latter would compensate for out-of-pocket expenses and lost time on agricultural and business activities, in their view.

## Discussion

The use of PDs in this study was a unique approach to determine the amount of time CDDs were allocating to their NTD Programme responsibilities in Uganda. The data show that to conduct NTD Programme activities a CDD spends, on average, two and a half working weeks per year. Subsequently the CDDs personal time, expenses and time available for work at home, in the shamba and other forms of livelihood are negatively affected. The CDDs that only have one package of drugs to deliver over the IPC campaign spent substantially less time (two weeks) on the NTD Programme than those who required three and a half weeks to deliver three rounds of treatment. Moreover, as CDDs took longer to deliver the treatment to targeted populations, their performance decreased. Mixed methods provided evidence to evaluate the ability and motivation of the CDDs to successfully and sustainably deliver an integrated programme for NTDs. Although the majority of CDDs would return to their role in the programme the following year, they were less motivated to participate in the NTD Programme than in other health interventions. Lack of incentives was a major contributor to feeling less motivated and undervalued.

There is a growing body of literature which highlights the importance of CDDs participation in the successful delivery and sustainability of PC for the NTDs and other health-related programmes such as home-based management of fever and Integrated Community Case Management [28–30]. Few, however, look at time spent by CDDs in distributing health packages [31]. Furthermore, the costs of CDD involvement in programmes is rarely valued nor included in programme economic evaluations [32, 33]. In this study, time allocated by the CDDs towards their NTD Programme duties were a significant factor affecting their performance. Unsurprisingly the number of households (on average 90 per CDD) and village population (on average 497 per CDD) served were associated with the amount of time spent of NTD Programme activities by the CDDs. Katabarwa and colleagues included the number of households served by a CDD in Uganda and Cameroon [31] and found that programme coverage improved when distributors served fewer households (< 20), worked within the kinship system and worked within a radius of 1 km of their activities. This study aligns with those findings and it is intuitive that if there are more households in a population then it will take longer to sensitise and treat all of them.

The number of drug delivery rounds over the IPC campaign for NTDs was also influential in how much time the CDDs spent on the programme. With fewer deliveries required by the CDDs, the time spent on NTD Programme duties was significantly lower. Information elucidated from the CDDs during the semi-structured interviews and FGD revealed that time spent on visits to collect drugs from health unit was increased with the number of drug delivery rounds expected of them i.e. one collection of drugs for each disease(s) targeted. In addition, for a fifth of respondents (21 %), inadequate drug forecasting resulted in insufficient quantities of drugs, at the health units, to reach target eligible population. CDDs thus returned frequently to the health units to check if more drugs had arrived from Kampala via the district headquarters. Consequently, the increased time spent on collecting drugs from health units, due to drug package or drug stock-outs, contributed to a poorer coverage performance by the CDDs. A further factor contributing to poorer performance could be the decreasing amount of time recorded on health education and mobilisation activities. These findings are in contrast to several studies where adding additional health interventions to the CDTI platform improved programme coverage [11, 34, 35] but, in agreement with the study in Cameroon and Uganda where the performance of the CDDs was compromised by added interventions [31].

Our study also estimated the opportunity costs of the CDDs involvement in the NTD Programme in Uganda. A limited number of studies have reported the opportunity costs of CDDs in health interventions [11, 24, 36]. The average two and half weeks volunteered by a CDD for NTD activities is equivalent to a salary of $35.71, based on local casual labour wages. For the same number of days a teacher would earn $49.06, a Health Assistant at a health centre would earn $75.19 and a District NTD Focal Point $121.11. When the average value of the CDDs opportunity cost is multiplied by the 61,000 CDDs that were trained under the NTD Programme that year (unpublished data, RTI International semi-annual report 2008/9), this amounts to US$2,178,310, which is a substantial contribution by the CDDs to the overall economic programme costs. In reality the CDDs received the equivalent of, on average, US$1.80 allowance for training and a t-shirt (US$1.86). If all were to receive a t-shirt, which was not the case in the study districts, the combined cost for allowance and t-shirts for the CDDs would be an estimated $223,260 which is a 10^th^ of the total value of the opportunity costs.

Similar to other studies [11, 23, 37], CDDs in Uganda are fully aware that they were committing to unpaid work and testified that they assume the responsibilities of the NTD Programme for reasons of intrinsic and extrinsic motivation. Nevertheless, this study highlights the significant amount of time forgone by the CDDs on their regular income or subsistence duties, the out-of-pocket expenses incurred and due to feeling undervalued, a better incentive package would be justified. In a recent study Downs et al. describe a framework where if work complexity is increased, so is the demand for incentives (monetary and non-monetary), and efforts towards meeting these demands can lead to an increased perceived value of the CDD position which can motivate CDDs to attaining a higher level of performance [38]. The use of pay for performance, or results-based financing, is being increasingly tested and used in developing countries [39–41]. These systems involve rewarding individuals for reaching their targets, such as numbers treated or programme coverage attained with financial or materials to effect an improvement in performance. In the context of the NTD Programme, however, such systems might only be feasible for CDDs if there was a general health system shift to such a reward scheme. Additional areas of concern for such schemes are false reporting and the lowering and even removal of intrinsic motivation in participants [39]. In contrast to these reward schemes, and perhaps more realistic for the NTD Programme, would be to employ alternative distribution mechanisms to what is currently in place. The traditional kinship system where each CDD treats below 20 households, which Katabarwa and colleagues have shown to be successful in terms of CDD performance, workload reduction and involvement in other health activities, in comparison to CDTI [22] offers one alternative distribution mechanism. In addition the demand for monetary incentives was reduced under the kinship system. Alternatively, the NTD Programme could choose to support and strengthen the existing health system through the VHT [7]. In Uganda one VHT member should serve approximately 25 to 30 households [7]. To support the VHT the NTD Programme would need to work closely with other MoH department to establish the needs of the VHTs in the target districts, for example, the provision or loan of bicycles and joint treatment registers and ensure reporting practices were standardised and in-line with those of the VHT [7]. Either of these distribution mechanisms would mean CDDs would be targeting less than a third of households than they are currently required to. Nonetheless, both suggested mechanisms would cause an increased cost to the programme. In the case of the kinship system the numbers of CDDs requiring training would approximately double and the VHT system may also require more training and the periodic supply of support. However, the potential for increased continuity, sustained performance and a decrease in the opportunity costs and out-of-pocket expenses incurred by the CDDs, could be argued to far outweigh the expense.

### Limitations

The main limitation of this study is that the teachers who distribute drugs for schistosomiasis and STH through a school-based system under the NTD Programme were not included. The teachers incur an opportunity cost for the time spent distributing the drugs and not teaching, however, as there is no loss of income by participating in the NTD Programme they were excluded from this study. The second limitation is that this study did not collect data on the number of drugs delivered to each district and the timeliness of their arrival. As the programme was in its second year and was using previous stand-alone programmes as a platform from which to base drug distribution mechanisms, poor drug logistics was not perceived to be an issue at the time of study design. Finally, the study would be strengthened if it had measured attrition rates in the CDDs. Community-based interventions are at-risk of high attrition rates in CDDs which, due to a lack of continuity, can undermine the effectiveness of programmes [42, 43].

## Conclusions

The availability, capacity, acceptance, and indeed ownership of the programme by the CDDs may only be one facet of sustainability of an NTD Programme, but it is critical for both effectiveness and longevity of the control and elimination efforts. The role of the CDD in the integrated NTD Programme requires a substantial commitment of time to achieve treatment goals. Additional workload is created with increasing number of delivery rounds within an IPC campaign and hampered by untimely national drug logistics. CDDs, subsequently, fail to achieve their target treatment coverage and there is limited access to these safe, effective and donated drugs for vulnerable individuals and families. For their performance, whether good or poor there is no feedback or consequence for the CDDs from the NTD Programme. Ultimately if programme coverage of 75 % and over is not being achieved as a minimum then the NTD Programme is not being effective and treatment will need to continue indefinitely and morbidity control and elimination goals for 2020 will not be reached. In order to utilise resources effectively and efficiently improved drug supplies and logistics to the districts and alternative support systems to the CDDs are imperative. This would involve investment in financial and non-financial resources yet would pay dividends in programme performance and sustainability. Affordable programmes with high impact are extremely desirable in the African context and continued high coverage of treatment with community participation and ownership are essential elements in their success.

## Abbreviations

ALB, albendazole; CDD, community drug distributor; CDTI, community directed treatment with ivermectin; IPC, integrated preventive chemotherapy; IVM, ivermectin; FGD, focus group discussion; PC, preventive chemotherapy; PD, pictorial diary; PZQ, praziquantel; NTD, neglected tropical diseases; WHO, World Health Organisation; VHT, village health teams; ZIT, azithromycin

## Additional file

Additional file 1: Figure S1. Hour Pictorial Diary. (PDF 390 kb)
