# Supplementary material for: A mixed methods approach to evaluating community drug distributor performance in the control of neglected tropical diseases
Source: Parasit Vectors. 2016 Jun 16;9:345. doi: 10.1186/s13071-016-1606-2 (PMC4910194; doi:10.1186/s13071-016-1606-2)
Supplement: Additional file 1: Figure S1. — Hour Pictorial Diary. (PDF 390 kb) [file 13071_2016_1606_MOESM1_ESM.pdf]

Hour Pictorial Diary

Date ……./………/……..	EKIRO		KUMAKYA					MUTTUNTU			OLWEGGULO				EKIRO				
Name ……………………	11 - 12	12 - 1	1 - 2	2 - 3	3 - 4	4 - 5	5 - 6	6 - 7	7 - 8	8 - 9	9 - 10	10 - 11	11 - 12	12 - 1	1 - 2	2 - 3	3 - 4	4 - 5	5 - 6
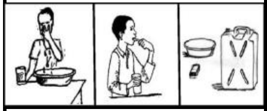																			
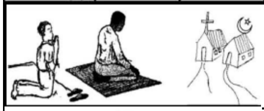																			
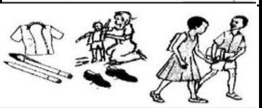																			
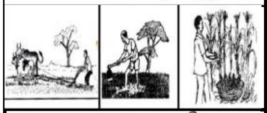																			
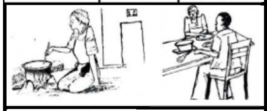																			
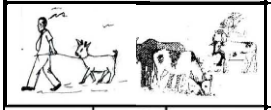																			
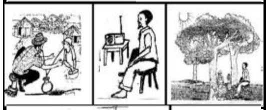																			
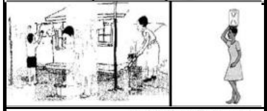																			
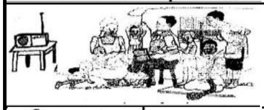																			
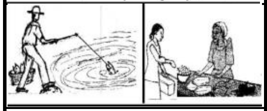																			
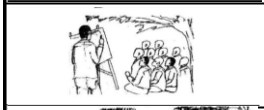																			
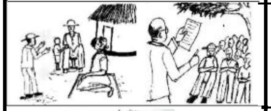																			
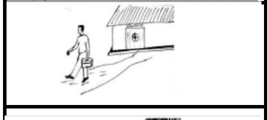																			
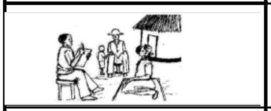																			
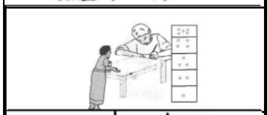																			
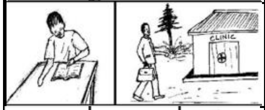																			
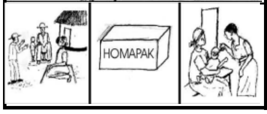																			
